# Supplementary material for: Vulnerability of Louisiana's coastal wetlands to present-day rates of relative sea-level rise
Source: Nat Commun. 2017 Mar 14;8:14792. doi: 10.1038/ncomms14792 (PMC5355890; doi:10.1038/ncomms14792)
Supplement: Supplementary Information — Supplementary Tables, Supplementary Figures and Supplementary References. [file ncomms14792-s1.pdf]

Supplementary Table 1. **Size of previously published rod-surface elevation table data sets.** The list below includes the number of rod-surface elevation tables (RSETs) used in 63 previously published studies. Our data set (n = 274) is an order of magnitude larger than the largest regionally contiguous RSET data set[20]. Supplementary Table 1 is an extension of previously published RSET data compilations[64,65].

| **Study/Reference #** | **Number of RSETs** | **Location** |
|---|---|---|
| | | **United States** |
| 1 | 6 | California |
| 2 | unspecified | California |
| 3 | 8 | California |
| 4 | 2 | Chesapeake Bay |
| 5 | 179 | East Coast (and parts of Europe) |
| 6 | 16 | Florida and the Carribbean |
| 7 | unspecified | Florida |
| 8 | unspecified | Florida |
| 9 | 9 | Florida |
| 10 | 9 | Florida |
| 11 | 2 | Louisiana |
| 12 | 18 | Louisiana |
| 13 | 6 | Louisiana |
| 14 | 9 | Louisiana |
| 11 | 10 | Louisiana |
| 15 | 20 | Louisiana |
| 16 | 3 | Louisiana |
| 17 | 6 | Maine |
| 18 | 6 | Maryland |
| 19 | 12 | Maryland |
| 20 | 25 | Maryland |
| 21 | 14 | Massachusetts, New Jersey, and Virginia |
| 22 | 1 | Mississippi Delta Region |
| 23 | 12 | Mississippi Delta Region |
| 24 | 6 | Mississippi Delta Region |
| 25 | 4 | Mississippi Delta Region |
| 26 | 14 | New Hampshire and Massachusetts |
| 27 | unspecified | New York |
| 28 | 3 | North Carolina |
| 29 | 2 | Oregon |
| 30 | 3 | South Carolina |
| 31 | 4 | Southern Region |
| 32 | 22 | Southern Region and Caribbean |
| 33 | 15 | Texas and Louisiana |

| | | |
|---|---|---|
| 34 | 12 | Texas |
| 35 | 10 | Washington DC |
| 36 | unspecified | Washington |
| | | |
| | | **Oceania** |
| 37 | 69 | Australia |
| 38 | 9 | Australia |
| 39 | 9 | Australia |
| 40 | 24 | Australia |
| 41 | 12 | Australia |
| 42 | 12 | Australia (Brisbane) |
| 43 | 6 | Australia (Minnamurra River) |
| 44 | 9 | Australia (Queensland) |
| 45 | 18 | Micronesia |
| 46 | 12 | New Zealand |
| 47 | 153 | Indo-Pacific Region |
| | | |
| | | **North and Central America** |
| 48 | 16 | Canada (Bay of Fundy) |
| 49 | 3 | Canada (Bay of Fundy) |
| 50 | 5 | Canada (Bay of Fundy) |
| 51 | 9 | Belize |
| 52 | 18 | Honduras |
| | | |
| | | **Europe** |
| 53 | 2 | France (Rhone Delta) |
| 54 | 20 | Italy (Venice Lagoon) |
| 55 | 10 | Italy (Venice Lagoon) |
| 56 | 4 | Italy (Venice Lagoon) |
| 57 | 6 | Spain (Ebro Delta) |
| 58 | 4 | Spain (Ebro Delta) |
| 59 | 55 | Spain, France, Italy |
| 60 | 13 | The Netherlands |
| 61 | 10 | United Kingdom |
| 62 | 8 | United Kingdom |
| 63 | 11 | United Kingdom |

Supplementary Table 2. **Rates of surface-elevation change, vertical accretion, and shallow subsidence by wetland type.**

| | Surface-elevation change rate (mm/yr) | | | Vertical accretion rate (mm/yr) | | | Shallow subsidence rate (mm/yr) | | | | |
|---|---|---|---|---|---|---|---|---|---|---|---|
| | **Mean** | **Median** | **s.d.** | **Mean** | **Median** | **s.d.** | **Mean** | **Median** | **s.d.** | **n** | **% of Total** |
| ***Total*** | 3.8 | 4.1 | 7.4 | 10.7 | 9.5 | 7.8 | 6.9 | 6.0 | 7.9 | 274 | 100% |
| *Fresh marsh* | 4.4 | 4.7 | 10.7 | 12.2 | 10.3 | 7.6 | 7.8 | 8.7 | 10.7 | 31 | 11% |
| *Intermediate marsh* | 2.5 | 2.0 | 7.5 | 9.9 | 9.5 | 6.3 | 7.4 | 7.1 | 5.8 | 83 | 30% |
| *Brackish marsh* | 3.0 | 3.5 | 5.4 | 8.7 | 8.6 | 4.5 | 5.7 | 5.3 | 4.8 | 74 | 27% |
| *Saline marsh* | 6.3 | 7.7 | 8.4 | 13.6 | 10.0 | 12.5 | 7.3 | 3.4 | 12.3 | 57 | 21% |
| *Swamp* | 4.1 | 3.9 | 3.4 | 10.5 | 9.5 | 4.7 | 6.4 | 6.1 | 4.9 | 29 | 11% |
| ***Mississippi Delta*** | 5.7 | 5.8 | 7.2 | 12.8 | 11.3 | 8.4 | 7.1 | 6.0 | 8.7 | 185 | 100% |
| *Fresh marsh* | 8.1 | 7.0 | 10.5 | 14.8 | 15.0 | 7.8 | 6.7 | 8.7 | 12.7 | 19 | 10% |
| *Intermediate marsh* | 5.4 | 5.1 | 7.2 | 13.7 | 12.7 | 6.2 | 8.2 | 8.0 | 4.8 | 42 | 23% |
| *Brackish marsh* | 4.6 | 4.4 | 4.8 | 10.6 | 10.3 | 4.1 | 5.9 | 5.5 | 4.3 | 45 | 24% |
| *Saline marsh* | 6.9 | 8.1 | 8.8 | 14.5 | 11.6 | 13.0 | 7.6 | 3.4 | 13.0 | 50 | 27% |
| *Swamp* | 4.1 | 3.9 | 3.4 | 10.5 | 9.5 | 4.7 | 6.4 | 6.1 | 4.9 | 29 | 16% |
| ***Chenier Plain*** | -0.2 | -0.5 | 6.3 | 6.3 | 5.9 | 3.7 | 6.5 | 5.8 | 6.3 | 89 | 100% |
| *Fresh marsh* | -1.6 | -3.0 | 8.4 | 8.0 | 7.5 | 5.0 | 9.6 | 9.8 | 6.7 | 12 | 13% |
| *Intermediate marsh* | -0.6 | -1.0 | 6.7 | 6.0 | 6.0 | 3.6 | 6.6 | 6.2 | 6.7 | 41 | 46% |
| *Brackish marsh* | 0.5 | 1.5 | 5.4 | 5.8 | 4.5 | 11.6 | 5.3 | 5.1 | 5.6 | 29 | 29% |
| *Saline marsh* | 1.7 | 1.3 | 2.8 | 7.0 | 6.3 | 3.5 | 5.3 | 3.7 | 4.1 | 7 | 8% |
| *Swamp* | N/A | N/A | N/A | N/A | N/A | N/A | N/A | N/A | N/A | 0 | 0% |

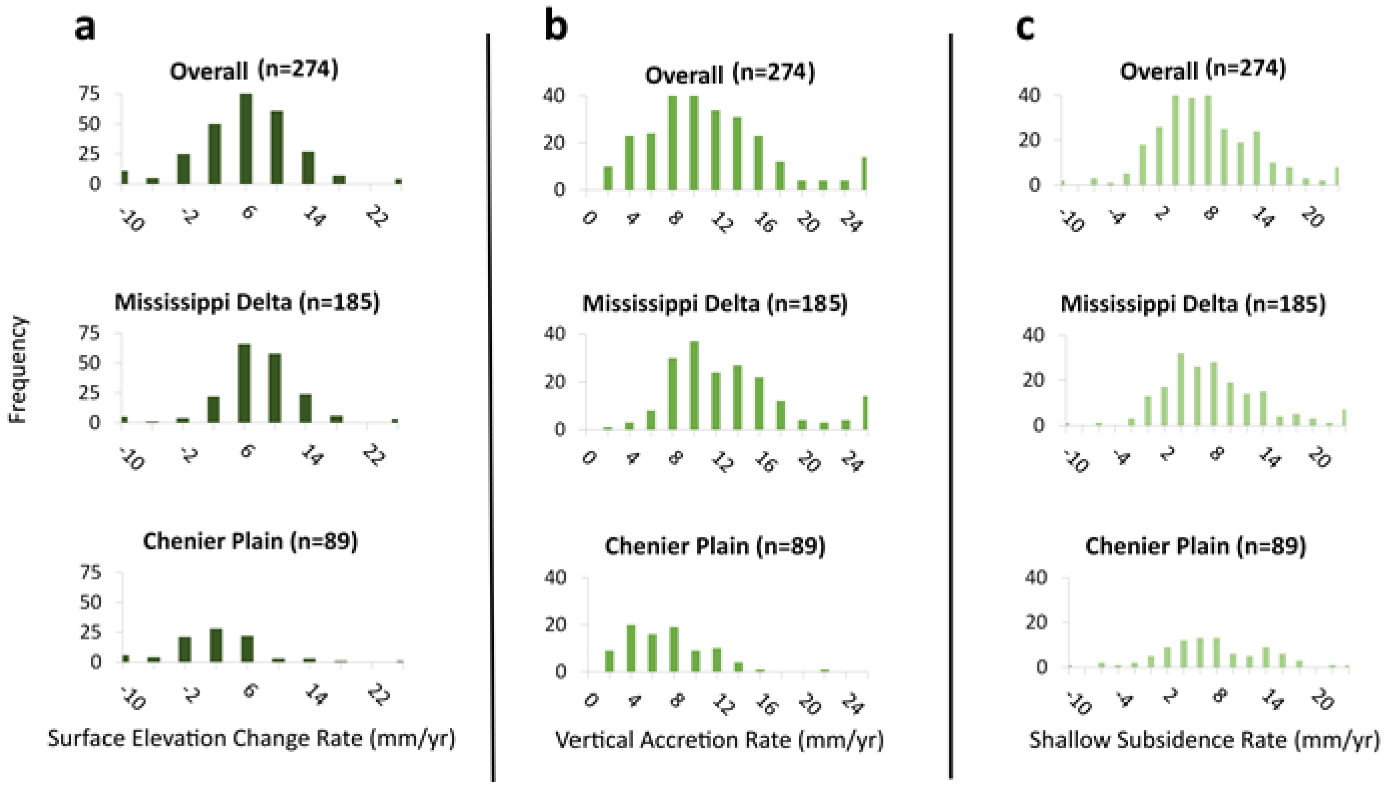


Supplementary Figure 1. **Frequency histograms with rates of (a) surface-elevation change, (b) vertical accretion, and (c) shallow subsidence.**

Supplementary Table 3. **GPS-measured and predicted deep subsidence rates in the Mississippi Delta.** Vertical velocity data were obtained from 13 GPS stations (data from Karegar et al., 2015) within the Mississippi Delta that were used to create a linear model (Fig. 4) of deep subsidence rates. Predicted vertical velocity is obtained by solving this linear equation as a function of latitude. Only GPS sites within the Mississippi Delta with ≥5 years of observation were included.

| GPS Station | Latitude | Longitude | Length of observation (yr) | GPS foundation depth (m) | GPS measured vertical velocity (mm/yr) | Predicted vertical velocity (mm/yr) | Difference between GPS-measured vs predicted vertical velocity (mm/yr) |
|---|---|---|---|---|---|---|---|
| BVHS | -89.41 | 29.34 | 11.93 | >20 | -5.7 | -5.27 | 0.43 |
| COVG | -90.10 | 30.48 | 10.02 | >15 | -0.8 | -1.04 | -0.24 |
| DSTR | -90.38 | 29.96 | 8.38 | unknown | -2.0 | -2.97 | -0.97 |
| ENG1 | -89.94 | 29.8 | 18.54 | ~3 | -2.3 | -3.26 | -0.96 |
| GRIS | -89.96 | 29.27 | 8.89 | unknown | -5.6 | -5.53 | 0.07 |
| HAMM | -90.47 | 30.51 | 13.45 | >15 | -1.0 | -0.92 | 0.08 |
| HOUM | -90.72 | 29.59 | 10.67 | >15 | -3.9 | -4.34 | -0.44 |
| LMCN | -90.66 | 29.25 | 11.26 | 36.5 | -6.5 | -5.61 | 0.89 |
| LWES | -90.35 | 29.90 | 6.67 | unknown | -2.7 | -3.19 | -0.49 |
| MSSC | -89.61 | 30.38 | 9.23 | unknown | -1.5 | -1.41 | 0.09 |
| NDBC | -89.61 | 30.36 | 13.18 | unknown | -1.3 | -1.48 | -0.18 |
| SJB1 | -91.11 | 30.40 | 5.36 | unknown | -1.5 | -1.33 | 0.17 |
| 1LSU | -91.18 | 30.41 | 11.21 | <15 | -2.9 | -1.30 | 1.60 |

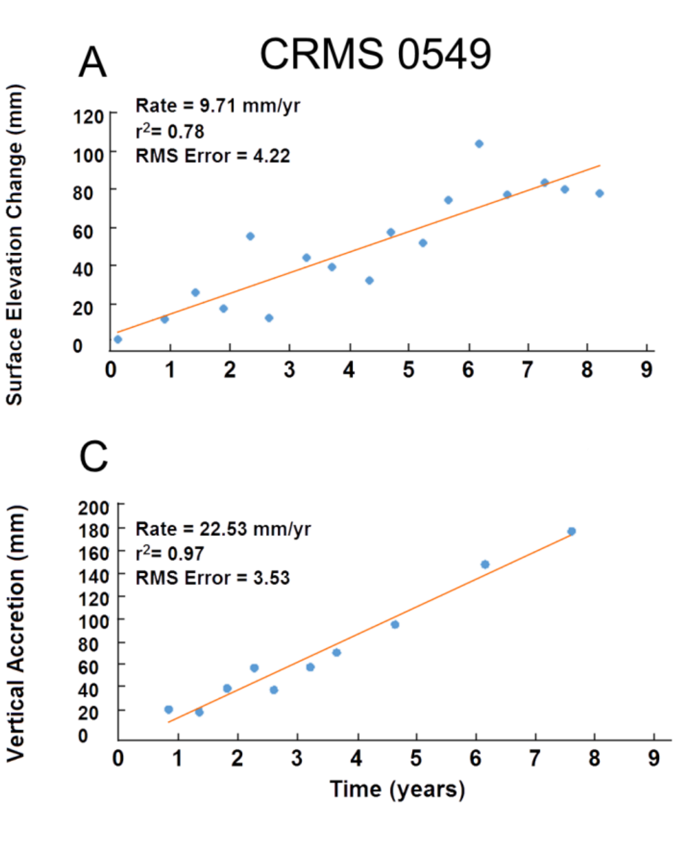


Supplementary Figure 2. **Methodology to determine rates of surface-elevation change and vertical accretion.** Following each site visit, individual measurements are averaged and plotted vs. time. A linear regression is carried out to determine the rate of surface-elevation change (a, c) and vertical accretion (b, d). For both SEC and VA records, mean site visit (i.e., static) measurements are indicated by the blue dots and the orange line indicates a linear regression analysis of the record which yields the eventual (i.e., long-term) SEC or VA rate. Short-term perturbations in the accretion history at individual CRMS sites contribute to within-site variability. It is important to note that due to the MH methodology, individual events that deviate from the overall trend of accretion at a CRMS site are necessarily time-averaged between site visits, and so the relative importance of individual events is dependent on the frequency of sampling.

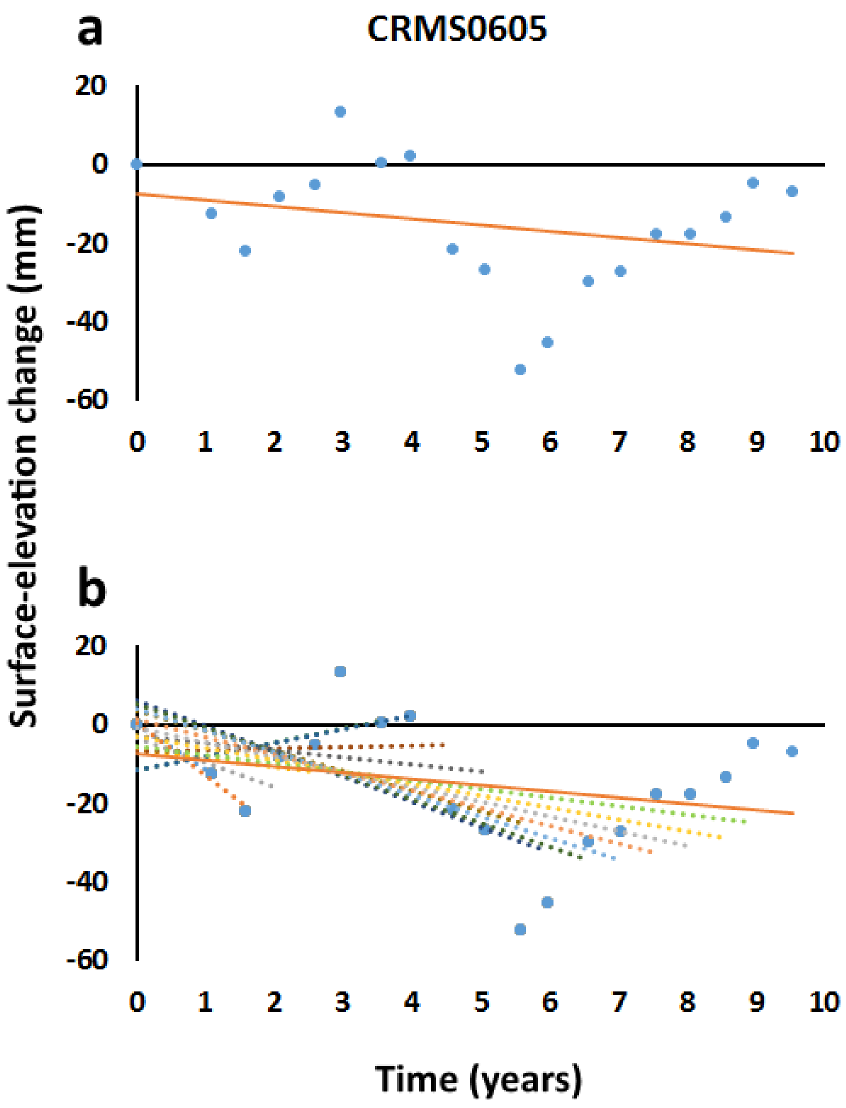


Supplementary Figure 3. **Comparison of eventual and site visit surface-elevation change linear regression analyses for site 0605.** The step-wise establishment of CRMS sites has resulted in variable durations of observation among sites. We determined the eventual (i.e., long-term) surface-elevation change rate for site 0605 with blue dots indicating site visit surface elevation measurements and the solid orange line indicating the linear regression (i.e., the eventual SEC rate) for this record (a), i.e., similar to Supplementary Fig. 2c. We then determined linear regressions for site visit SEC rates at each time step (i.e., approximately every six months through the duration of observation) with each of the dotted lines representing a site visit SEC rate of different length and the solid orange line representing the eventual SEC rate (b). Short-term site visit SEC rates (≤4 years of observation) produce highly variable campaign SEC rates that may deviate significantly from the eventual SEC rate. Longer-term site visit SEC rates (≥5 years of observation) approximate the eventual SEC rate.

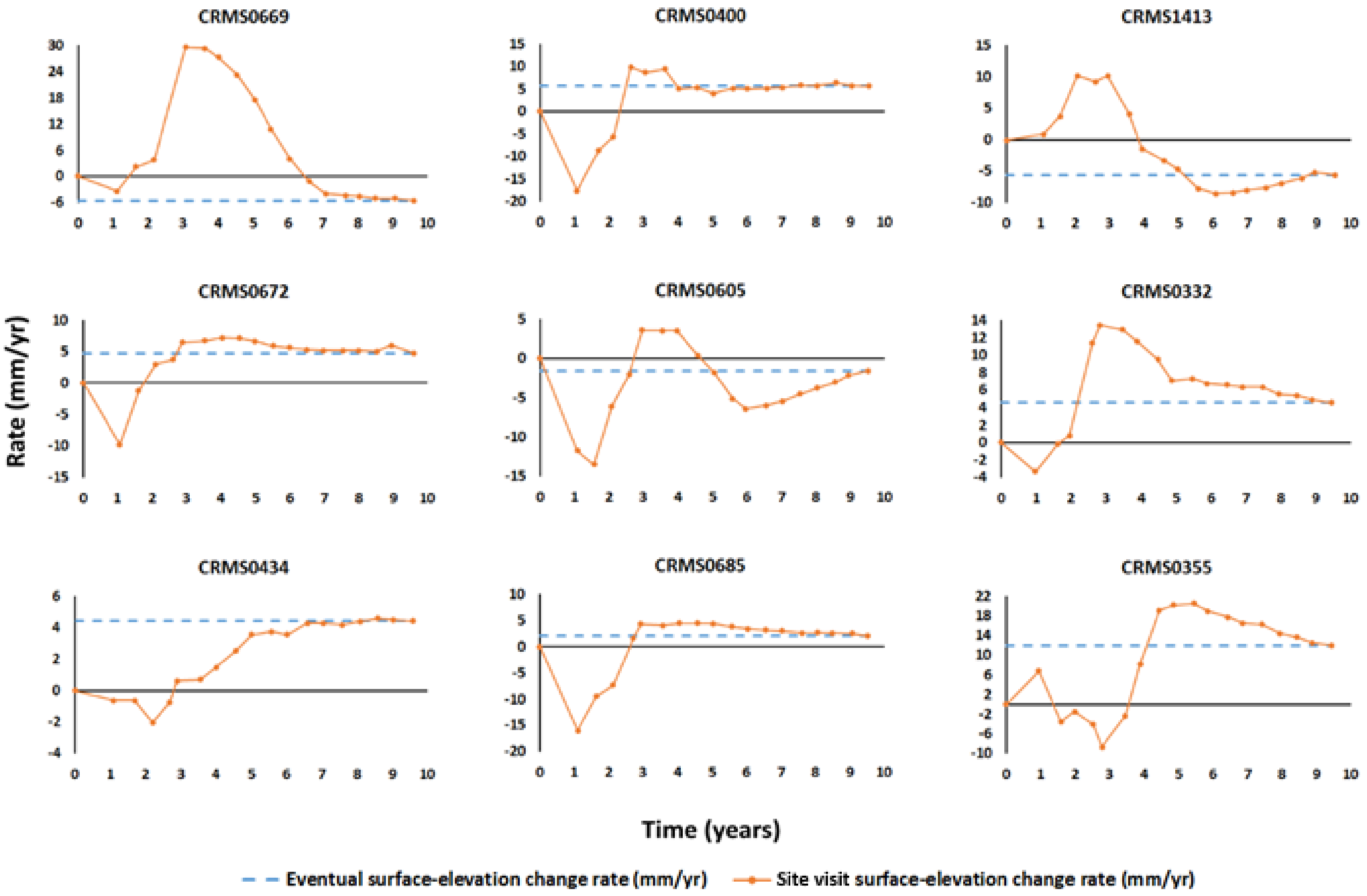


Supplementary Figure 4. **Comparison of eventual and site visit surface-elevation change for the 9 longest records.** The difference between each site visit SEC rate (orange line) and the eventual SEC rate (dashed blue line) is plotted for the 9 sites with the longest duration of observation. The difference at each time step is tracked over the full duration of observation for each of the 9 selected sites. We find that in most cases, the difference between the site visit SEC rate and the eventual SEC rate approaches its minimum after 5 years of observation (and sometimes earlier). All sites included in this analyses have been monitored for 6 years or more. It is important to note that this analysis fundamentally assumes that SEC rates at a given site approach a "true" value over time and that these rates are appropriately represented by a linear relationship as time progresses. While this is not necessarily correct, it constitutes a more stringent assessment of required length of observation record than what has commonly been used[47].

Supplementary Table 4. **Effect of removing noisy surface-elevation change and vertical accretion records.** Descriptive statistics for rates of surface-elevation change and vertical accretion and for the Mississippi Delta and Chenier Plain sub-regions were calculated using the full data set as well as a smaller, reduced error data set. We compare the descriptive statistics of these two data sets to evaluate the effect that noisy data may have on the results. In the reduced error data set, sites with a root mean squared (RMS) error in the $90^{th}$ percentile (i.e., the noisiest records) for surface-elevation change and/or vertical accretion were removed. Removal of high RMS error records generally removed exceptionally low minimum and/or high maximum values and lowered the standard deviation.

| | **Full data set n=274** | **Reduced error data set n=227** | **Full data set n=274** | **Reduced error data set n=227** |
|---|---|---|---|---|
| | **Overall** | | | |
| | *Surface-elevation change rate (mm/yr)* | | *Vertical accretion rate (mm/yr)* | |
| Mean | 3.8 | 3.7 | 10.7 | 10.5 |
| Median | 4.1 | 4.1 | 9.5 | 9.5 |
| Standard deviation | 7.4 | 6.0 | 7.8 | 7.8 |
| Minimum | -41.0 | -18.0 | 0.2 | 0.9 |
| Maximum | 46.0 | 31.9 | 83.7 | 83.7 |
| | **Mississippi Delta** | | | |
| | *Surface-elevation change rate (mm/yr)* | | *Vertical accretion rate (mm/yr)* | |
| Mean | 5.7 | 5.6 | 12.8 | 12.6 |
| Median | 5.8 | 5.8 | 11.3 | 11.2 |
| Standard deviation | 7.2 | 5.1 | 8.4 | 8.5 |
| Minimum | -41.0 | -18.9 | 1.6 | 2.0 |
| Maximum | 46.0 | 31.9 | 83.7 | 83.7 |
| | **Chenier Plain** | | | |
| | *Surface-elevation change rate (mm/yr)* | | *Vertical accretion rate (mm/yr)* | |
| Mean | -0.2 | -0.9 | 6.3 | 6.1 |
| Median | -0.5 | -0.6 | 5.9 | 5.5 |
| Standard deviation | 6.3 | 5.1 | 3.7 | 3.4 |
| Minimum | -17.3 | -17.3 | 0.2 | 0.9 |
| Maximum | 22.5 | 12.2 | 20.6 | 14.7 |

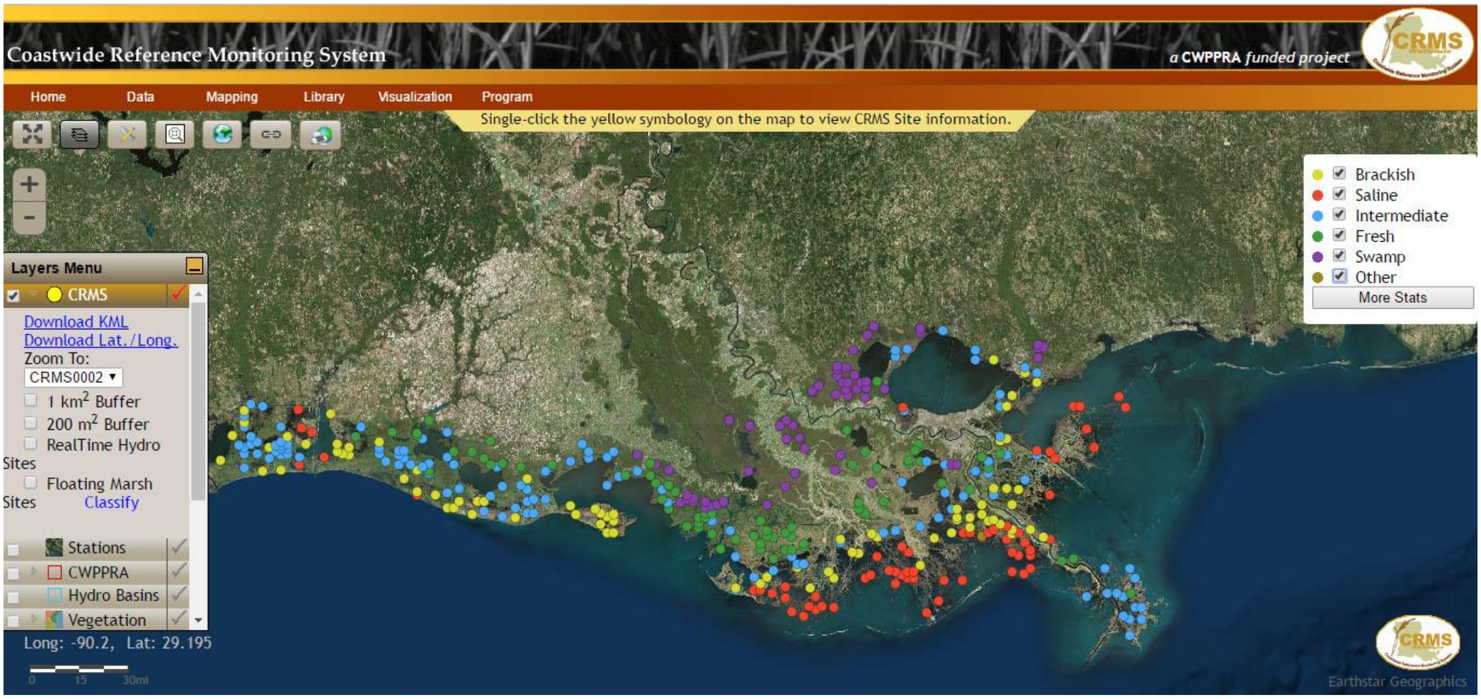


Supplementary Figure 5. **Distribution of wetland types in coastal Louisiana.** Data are taken from the Coastwide Reference Monitoring System[66] and wetland types are based upon an established classification system[67]. Here all 391 CRMS sites (including the 274 sites used in this study) are represented.

Supplementary References


1. Boumans, R., M. Ceroni, D. Burdick, D. Cahoon, & C. Swarth. Sediment elevation dynamics in tidal marshes: Functional assessment of accretionary biofilters. CICEET Final Report for the period of 8/15/1999 through 8/15/2002. Cooperative Institute for Coastal and Estuarine Environmental Technology, Durham, New Hampshire (2003).

2. Reed, D.J. Understanding tidal marsh sedimentation in the Sacramento-San Joaquin Delta, California. *J. Coastal Res.* **36,** 605-611 (2002).

3. Wallace, K., Callaway, J., & Zedler, J. Evolution of tidal creek networks in a high sedimentation environment: A 5-year experiment at Tijuana Estuary, California. *Estuaries* **28,** 795-811 (2005).

4. Rooth, J., & Stevenson, J. Sediment deposition patterns in Phragmites australis communities: Implications for coastal areas threatened by rising sea-level. *Wetl. Ecol. Manag.* **8,** 173-183 (2000).

5. Kirwan, M.L., Temmerman, S., Skeehan, E., Guntenspergen, G., & Fagherazzi, S. Overestimation of marsh vulnerability to sea level rise. *Nat. Clim. Change* **6,** 253-260 (2016).

6. McKee, K.L. Biophysical controls on accretion and elevation change in Caribbean mangrove ecosystems. *Estuar. Coast. Shelf Sci.* **91,** 475–483 (2011).

7. Cahoon, D. R. & Lynch, J. C. Vertical accretion and shallow subsidence in a mangrove forest of southwestern Florida, USA. *Mangroves Salt Marshes* **1**, 173–186 (1997).

8. Hendrickson, J. C. Coastal wetland response to rising sea-level: quantification of short- and longterm accretion and subsidence, northeastern Gulf of Mexico. MS Thesis, Florida State University, Tallahassee, FL (1997).

9. Whelan, K.R.T., Smith, T.J., Cahoon, D.R., Lynch, J.C., & Anderson, G.H. Groundwater control of mangrove surface elevation: shrink-swell of mangrove soils varies with depth. *Estuaries* **28,** 833–843 (2005).

10. Whelan, K.R.T., Smith, T.J., Anderson, G.H., & Ouellette, M.L. Hurricane Wilma's impact on overall soil elevation and zones within the soil profile in a mangrove forest. *Wetlands* **29,** 16-23 (2009).

11. Cahoon, D.R., White, D.A. & Lynch, JC. Sediment infilling and wetland formation dynamics in an active crevasse splay of the Mississippi River delta. *Geomorphology* **131,** 57–68 (2011).

12. Lane, R.R., Day, J.W., & Day, J.N. Wetland surface elevation, vertical accretion, and subsidence at three Louisiana estuaries receiving diverted Mississippi River water. *Wetlands* **26,** 1130-1142 (2006).

13. Rybczyk, J. M., & Cahoon, D.R. Estimating the potential for submergence for two subsiding wetlands in the Mississippi River delta. *Estuaries* **25,** 985-998 (2002).

14. Boumans, R. M. J., Day, J. W. & Kemp G. P. The effect of intertidal sediment fences on wetland surface elevation, wave energy and vegetation establishment in two Louisiana coastal marshes. *Ecol. Eng.* **9,** 37–50 (1997).

15. Ford, M. A. & Grace, J. B. Effects of vertebrate herbivores on soil processes, plant biomass, litter accumulation and soil elevation changes in a coastal marsh. *J. Ecol.* **86,** 974-982 (1998).

16. Guntenspergen, G., et al. Disturbance and recovery of the Louisiana coastal marsh landscape from the impacts of Hurricane Andrew. *J. of Coastal Res.* SI **21,** 324-339 (1995).

17. Bowron, T., Neatt, N., van Proosdij, D., Lundholm, J. & Graham, J. Macro-tidal salt marsh ecosystem response to culvert expansion. *Restor. Ecol.* **19,** 307–322 (2011).

18. Kroes, D. E. & Hupp, C.R. The effect of channelization on floodplain sediment deposition and subsidence along the Pocomoke River, Maryland. *J. Am. Water Resour. Assoc*. **46,** 686-699 (2010).

19. Delgado, P. Hensel, P.F., Swarth, C.W., Ceroni, M., and Boumans, R. Sustainability of a tidal freshwater marsh exposed to a long-term hydrologic barrier and sea level rise. *Estuar. Coast.* **36,** 585-594 (2013).

20. Childers, D. L., Sklar, F. H., Drake, B. & Jordan, T. Seasonal movements of sediment elevation in three mid-Atlantic estuaries. *J. Coastal Res.* **9,** 986-1003 (1993).

21. Erwin, R. M., Cahoon, D. R., Prosser, D. J., Sanders, G. M. & Hensel, P. Surface elevation dynamics in vegetated Spartina marshes versus unvegetated tidal ponds along the mid-Atlantic coast, USA, with implications to wetlands. *Estuar. Coast.* **29,** 96-108 (2006).

22. Cahoon, D. R., Marin, P. E., Black, B. K. & Lynch, J. C. A method for measuring vertical accretion, elevation, and compaction of soft, shallow-water sediments. *J. Sediment. Res.* **70,** 1250- 1253 (2000).

23. Day, J. W., et al. Vegetation death and rapid loss of surface elevation in two contrasting Mississippi delta salt marshes: the role of sedimentation, autocompaction and sea-level rise. *Ecol. Eng.* **37,** 229-240 (2011).

24. Ford, M. A., Cahoon, D. A. & Lynch, J. C. Restoring marsh elevation in a rapidly subsiding salt marsh by thin-layer deposition of dredged material. *Ecol. Eng.* **12,** 189-205 (1999).

25. McKee, K. L. & Cherry, J. A. Hurricane Katrina sediment slowed elevation loss in subsiding brackish marshes of the Mississippi River delta. *Wetlands* **29,** 2-15 (2009).

26. Boumans, R. M. J., Burdick, D. M. & Dionne, M. Modeling habitat change in salt marshes after tidal restoration. *Restor. Ecol.* **10,** 543-555. (2002)

27. Roman, C. T., King, J. W., Cahoon, D. R., Lynch, J. C. & Appleby, P. G. Evaluation of marsh development processes at Fire Island National Seashore (New York): recent and historic perspectives. Technical Report NPS/NER/NRTR – 2007/089, National Park Service, Boston MA (2007).

28. Mattheus, C. R., Rodriguez, A. B., McKee, B. A. & Currin, C. A. Impact of land-use change and hard structures on the evolution of fringing marsh shorelines. *Estuar. Coast. Shelf S.* **88,** 365-376 (2010).

29. Cornu, C. E. & Sadro, S. Physical and functional responses to experimental marsh surface elevation manipulation in Coos Bay's South Slough. *Restor. Ecol.* **10,** 474-486 (2002).

30. Morris, J. T., Sundareshwar, P. V., Nietch, C. T., Kjerfve, B. & Cahoon, D. R. Responses of coastal wetlands to rising sea level. *J. Ecol.* **83,** 2869-2877 (2002).

31. Cahoon, D. R., Reed, D. J. & Day, J. W. Estimating shallow subsidence in microtidal salt marshes of the southeastern United States: Kaye and Barghoorn revisited. *Mar. Geol.* **128,** 1-9 (1995).

32. Cahoon, D. R. A review of major storm impacts on coastal wetland elevations. *Estuar. Coast.* **29,** 889-898 (2008).

33. Cahoon, D. R., Perez, B. C. Segura, B. D. & Lynch, J. C. Elevation trends and shrink-swell response of wetland soils to flooding and drying. *Estuar. Coast. Shelf S.* **91,** 463-474 (2011).

34. Cahoon, D. R., Ford, M. A. & Hensel, P. F. Ecogeomorphology of Spartina patens-dominated tidal marshes: solid organic matter accumulation, marsh elevation dynamics, and disturbance. In The Ecogeomorphology of Tidal Marshes, Coastal Estuarine Studies 59 (eds Fagherazzi, S., Marani, M. & Blum L. K.) 247-266 (American Geophysical Union, Washington, D. C., 2004).

35. Baldwin A.H., Hammerschlag R.S., & Cahoon D.R. Evaluation of restored tidal freshwater wetlands. In: Perillo GME, Wolanski E, Cahoon DR & Brinson MM (eds) Coastal wetlands: an integrated ecosystem approach, pp. 801-831. Elsevier, Oxford, UK (2009).

36. Cahoon, D. R., Day, J. W., Jr., & Reed, D. J. The influence of surface and shallow subsurface soil processes on wetland elevation: a synthesis. *Curr. Top.Wetland Biogeochemistry* **3,** 72-88 (1999).

37. Rogers, K., Wilton, K. M. & Saintilan, N. Vegetation change and surface elevation dynamics in estuarine wetlands of southeast Australia. *Estuar. Coast. Shelf S.* **66,** 559-569 (2006).

38. Rogers, K., Saintilan, N., Howe, A. J. & Rodríguez, J. F. (2013). Sedimentation, elevation and marsh evolution in a southeastern Australian estuary during changing climatic conditions. *Estuar. Coast. Shelf S.* **133,** 172-181.

39. Rogers, K., Saintilan, N. & Cahoon, D. Surface elevation dynamics in a regenerating mangrove forest ad Homebush Bay, Australia. *Wetlands Ecol. Manag.* **13,** 587-598 (2005).

40. Rogers, K., Saintilan, N. & Heinjnis, H. Mangrove encroachment of salt marsh in Port Bay, Victoria: the role of sedimentation, subsidence and sea level rise. *Estuaries* **28,** 551-559 (2005).

41. Howe, A. J., Rodríguez, J. F. & Saco, P. M. Surface evolution and carbon sequestration in disturbed and undisturbed wetland soils of the Hunter estuary, southeast Australia. *Estuar. Coast. Shelf S.* **84,** 75-83 (2009).

42. Lovelock, C. E., Bennion, V., Grinham, A. & Cahoon, D. R. The role of surface and subsurface processes in keeping pace with sea level rise in intertidal wetlands of Moreton Bay, Queensland, Australia. *Ecosystems* **14,** 745-757 (2011).

43. Oliver, T.S.N., Rogers, K., Chafer, C.J., & Woodroffe, C.D. Measuring, mapping and modelling: an integrated approach to the management of mangrove and saltmarsh in the Minnamurra River estuary, southeast Australia. *Wetl. Ecol. Manag.* **20,** 353–71 (2012).

44. Lovelock, C.E., et al. Contemporary rates of carbon sequestration through vertical accretion of sediments in mangrove forests and saltmarshes of South East Queensland, Australia. *Estuar. Coast.* **37,** 763 -771 (2014).

45. Krauss, K.W., et al. Surface elevation change and susceptibility of different mangrove zones to sea-level rise on Pacific high islands of Micronesia. *Ecosystems* **13,** 129-143 (2010).

46. Stokes, D. J., Healy, T. R. & Cooke, P. J. Surface elevation changes and sediment characteristics of intertidal surfaces undergoing mangrove expansion and mangrove removal, Waikaraka Estuary, Tauranga Harbour, New Zealand. *Int. J. Ecol. Dev.* **12,** 88-106 (2009).

47. Lovelock, C. E., et al. The vulnerability of Indo-Pacific mangrove forests to sea-level rise. *Nature* **526,** 559–565 (2015).

48. Pacquette, C. H., Sundberg, K. L., Boumans, R. M. J. & Chmura, G. L. Changes in saltmarsh surface elevation due to variability in evapotranspiration and tidal flooding. *Estuaries* **27,** 82-89 (2004)

49. Van Proosdij, D., Ollerhead, J. & Davidson-Arnott, R. G. D. Seasonal and annual variations in the volumetric sediment balance of a macro-tidal salt marsh. *Mar. Geol.* **225,** 103-127 (2006).

50. Van Proosdij, D., Lundholm, J., Neatt, N., Bowron, T. & Graham, J. Ecological re-engineering of a freshwater impoundment for salt marsh restoration in a hypertidal system. *Ecol. Eng.* **36,** 1314-1332 (2010).

51. McKee, K. L., Cahoon, D. R. & Feller, I. C. Caribbean mangroves adjust to rising sea level through biotic controls on change in soil elevation. *Global Ecol. Biogeogr.* **16,** 545-556 (2007).

52. Cahoon, D. R., et al. Mass tree mortality leads to mangrove peat collapse at Bay Islands, Honduras after Hurricane Mitch. *J. Ecol.* **91,** 1093-1105 (2003).

53. Hensel, P. E., Day, J. W. & Pont, D. Wetland vertical accretion and soil elevation change in the Rhône River delta, France: the importance of river flooding. *J. Coastal Res.* **15,** 668-681 (1999).

54. Day, J. W., et al. Soil accretionary dynamics, sea-level rise and the survival of wetlands in Venice lagoon: a field and modelling approach. *Estuar. Coast. Shelf S.* **49,** 607-628 (1999).

55. Day, J. W., Rismondo, A., Scarton, F., Are, D. & Cecconi, G. Relative sea level rise and Venice lagoon wetlands. *J. Coastal Conserv.* **4,** 27-34 (1998).

56. Scarton, D., Day, J. W., Rismondo, A., Cecconi, G. & Are, D. Effects of an intertital sediment fence on sediment elevation and vegetation distribution in a Venice (Italy) lagoon salt marsh. *Ecol. Eng.* **16,** 223-233 (2000).

57. Ibáñez, C., Sharpe, P. J., Day, J. W., Day, J. N. & Prat, N. Vertical accretion and relative sea level rise in the Ebro delta wetlands (Catalonia, Spain). *Wetlands* **30,** 979-988 (2010).

58. Ibañez, C., Antoni, C., Day, J. W. & Curcó, A. Morphologic development, relative sea level rise and sustainable management of water and sediment in the Ebre Delta, Spain. *J. Coastal Conserv.* **3,** 191-202 (1997).

59. Day, J.W, et al. Sustainability of Mediterranean deltaic and lagoon wetlands with sea-level rise: the importance of river input. *Estuar.Coast.* **34,** 483-493 (2011).

60. Vandenbruwaene, W., et al. Sedimentation and response to sea-level rise of a restored marsh with reduced tidal exchange: comparison with a natural tidal marsh. *Geomorphology* **130,** 115-126 (2011).

61. Cahoon, D. R., French, J. R., Spencer, T., Reed, D. & Moller. Vertical accretion versus elevational adjustment in UK saltmarshes: an evaluation of alternative methodologies. In Coastal and Estuarine Environments: sedimentology, geomorphology and geoarchaeology (eds. Pye, K. & Allen, J. R. L) 223-238 (Geological Society, London, 2000).

62. French, J. R. & Burningham, H. Tidal marsh sedimentation versus sea-level rise: A Southeast England estuarine perspective. Proceedings Coastal Sediments '03, Clearwater, Florida (2003).

63. Spencer, T., et al. Surface elevation change in natural and re-created intertidal habitats, eastern England, UK, with particular reference to Frieston Shore. *Wetlands Ecol. Manag.* **20,** 9-33 (2012).

64. Cahoon, D. Estimating relative sea-level rise and submergence potential at a coastal wetland. *Estuar. Coast.* **38,** 1077-1084 (2015).

65. Webb, E., et al. A global standard for monitoring coastal wetland vulnerability to accelerated sea-level rise. *Nat. Clim. Change.* **3,** 458 -465 (2013).

66. Coastal Protection and Restoration Authority (CPRA) of Louisiana. 2016. Coastwide Reference Monitoring System-Wetlands Monitoring Data. Retrieved from Coastal Information Management System (CIMS) database. http://cims.coastal.louisiana.gov. Accessed 04/15/2016.

67. Visser, J. M., Sasser, C. E., Chabreck, R. H. & Linscombe, R. G. The impact of a severe drought on the vegetation of a subtropical estuary. *Estuaries* **25,** 1184–1195 (2002).
